# Is there any association between National Institute of Health category IV prostatitis and prostate-specific antigen levels in patients with low-risk localized prostate cancer?

**DOI:** 10.1590/S1677-5538.IBJU.2015.0082

**Published:** 2016

**Authors:** Omer Gokhan Doluoglu, Cavit Ceylan, Fatih Kilinc, Eymen Gazel, Berkan Resorlu, Oner Odabas

**Affiliations:** 1 Department of Urology Clinic, Ankara Training and Research Hospital, , Ankara Turkey; 2 Department of Urology Clinic of Yüksek Ihtisas Training and Research Hospital, Ankara, Turkey

**Keywords:** Prostatitis, Prostatic Neoplasms, prostate-specific antigen (146–154) [Supplementary Concept]

## Abstract

**Purpose:**

We investigated the association between National Institute of Health category IV prostatitis and prostate-specific antigen levels in patients with low-risk localized prostate cancer.

**Materials and Methods:**

The data of 440 patients who had undergone prostate biopsies due to high PSA levels and suspicious digital rectal examination findings were reviewed retrospectively. The patients were divided into two groups based on the presence of accompanying NIH IV prostatitis. The exclusion criteria were as follows: Gleason score>6, PSA level>20ng/mL, >2 positive cores, >50% cancerous tissue per biopsy, urinary tract infection, urological interventions at least 1 week previously (cystoscopy, urethral catheterization, or similar procedure), history of prostate biopsy, and history of androgen or 5-alpha reductase use. All patient's age, total PSA and free PSA levels, ratio of free to total PSA, PSA density and prostate volume were recorded.

**Results:**

In total, 101 patients were included in the study. Histopathological examination revealed only PCa in 78 (77.2%) patients and PCa+NIH IV prostatitis in 23 (22.7%) patients. The median total PSA level was 7.4 (3.5–20.0) ng/mL in the PCa+NIH IV prostatitis group and 6.5 (0.6–20.0) ng/mL in the PCa group (p=0.67). The PSA level was≤10ng/mL in 60 (76.9%) patients in the PCa group and in 16 (69.6%) patients in the PCa+NIH IV prostatitis group (p=0.32).

**Conclusions:**

Our study showed no statistically significant difference in PSA levels between patients with and without NIH IV prostatitis accompanying PCa.

## INTRODUCTION

Prostate-specific antigen (PSA) is the most practical tumor marker used in the diagnosis of prostate cancer (PCa). Measurement of PSA levels has marked a new era in the diagnosis of PCa (1). PSA is a serine protease that is produced almost exclusively by the epithelial cells of the prostate. PSA is not a cancer-specific marker; rather, it is organ-specific (2). Therefore, its serum levels may increase in nonmalignant conditions such as benign prostate hypertrophy (BPH) and prostatitis. The histological architecture of the prostate is disturbed in both PCa and prostatitis, causing greater PSA leakage from the lumen of the prostatic glands into the circulation, increasing PSA levels.

The incidence of clinically insignificant PCa has increased along with the rise in the frequency of PSA screening and multicore biopsy. Therefore, many patients with PCa may undergo unnecessary definitive treatment. Conservative treatment options such as active surveillance have been proposed to decrease the incidence of overtreatment in this subgroup of patients. The risk classification system for localized PCa described by D'Amico et al. (3), which defines low risk as a PSA level<10ng/mL, Gleason score≤6, and clinical stage of T1 to T2a, is one of the most frequently used risk classification systems to determine the need for active surveillance. The new National Comprehensive Cancer Network (NCCN) guidelines (2014 version 2) clinically divide localized PCa into four groups based on pathological results and clinical parameters: very low risk, low risk, intermediate risk, and high risk. In addition to the classification by D'Amico et al. (3), the NCCN classification adds a very-low-risk group defined as a PSA density (PSAD) <0.15ng/mL/g, fewer than three positive prostate biopsy cores, and≤50% cancerous tissue in any core. The criteria added to the low-risk group are a clinical stage of T1 to T2a, Gleason score≤6, and PSA<10ng/mL. Active surveillance is included in the treatment protocol in both groups, and the treatment can be tailored according to the patient's life expectancy (4).

Several studies have shown that active surveillance is associated with very low progression and cancer-specific death rates in selected low-risk patients (5-7). This treatment option seems rational in low-risk patients with a life expectancy of>10 years.

National Institute of Health (NIH) category IV (asymptomatic) prostatitis is described as the presence of inflammatory cells in the biopsy specimen secondary to a high PSA level in an asymptomatic patient (8-10). Considering that most patients with newly diagnosed PCa are in stage T1c and that asymptomatic prostatitis increases the PSA level, one wonders whether the addition of prostatitis to PCa further increases the PSA level. If this is the case, patients in the NCCN very-low-risk and low-risk groups may be reclassified in the intermediate-risk group only because of a PSA level>10ng/mL, and their treatment strategy may change completely. Thus, we investigated the effects of concurrent NIH IV prostatitis on PSA levels in patients with low-risk localized PCa.

## MATERIALS AND METHODS

After approval from the local ethics committee, the data of 440 patients who had undergone prostate biopsies due to high PSA levels and suspicious digital rectal examination (DRE) findings in our hospital from 2012 to 2014 were reviewed retrospectively. The patients were divided into two groups according to the presence of accompanying NIH IV prostatitis. The exclusion criteria were as follows: Gleason score>6, PSA level>20ng/mL, >2 positive cores, >50% cancerous tissue per biopsy, urinary tract infection, urological interventions at least 1 week previously (cystoscopy, urethral catheterization, or similar procedures), a history of prostate biopsy, and a history of androgen or 5-alpha reductase use.

The patient's age, total PSA (tPSA) and free (fPSA) levels, f/tPSA, PSAD, and prostate volume (PV) were recorded. PSA was measured at least three times in all patients. The last pre-biopsy PSA measurement was used as the final value. Transrectal ultrasonography (TRUS)-assisted systematic 12-core prostate biopsy was performed in all patients. TRUS was performed using a LOG-13, 41123WS1 ultrasonography system (General Electric) with a 6.5-MHz biplane transrectal probe. The patients were given prophylactic antibiotics 1 day before biopsy. PV was calculated using TRUS and the ellipsoid formula (height×width×length×0.52). The histopathological preparations were examined again for NIH IV prostatitis by an experienced uropathologist in the pathology clinic of our hospital. NIH IV prostatitis was determined by the presence of inflammatory cell infiltration in the prostate biopsy specimen in the absence of symptoms.

The data analysis was performed using SPSS for Windows, version 11.5 (SPSS Inc., Chicago, IL, United States). Descriptive statistics for nominal variables and variables with a non-normal distribution are shown as the numbers of cases and (%), and the medians (min–max), respectively. The significance of differences in median values between the groups was investigated with the Mann–Whitney U test. Nominal variables were assessed by Pearson's chi-squared or Fisher's exact test. A p value<0.05 was considered statistically significant.

## RESULTS

In total, 101 patients fulfilled the inclusion criteria and were included in the study. Histopathological examination revealed PCa only in 78 (77.2%) patients and PCa+NIH IV prostatitis in 23 (22.8%) patients. The median patient age was 66 (43–76) years in the PCa group and 67 (59–76) years in the PCa+NIH IV prostatitis group (p=0.45). The median tPSA level was 7.4 (3.5–20.0) ng/mL in the PCa+NIH IV prostatitis group and 6.5 (0.6–20.0) ng/mL in the PCa group. Although the median PSA level was higher in the PCa+NIH IV prostatitis group, the difference between the groups was not statistically significant (p=0.67).The median PV was 35 (15–98) mL in the PCa group and 40 (20–90) mL in the PCa+NIH IV prostatitis group (p=0.03). The difference between the groups was statistically significant. The data of the two groups are presented in Table-1. The PSA level was ≤10ng/mL in 60 (76.9%) patients in the PCa group and in 16 (69.6%) patients in the PCa+NIH IV prostatitis group (p=0.32). The difference between the groups was not statistically significant. In PCa group, 22 of 78 (28.2%) patients were very low risk patients, and 38 (48.7%) of them were low risk patients. There were 10 (43.5%) patients with very low risk, and 6 (26.1%) patients with low risk in PCa+NIH prostatitis group (p=0.15). Cancer was detected in one core in 49 of 78 (62.8%) patients in PCa group, in the same way there was tumor in one core in 18 of 23 (66.3%) patients in PCa+NIH IV prostatitits group (p=0.16). The mean percent of tumor in the cores was 20%±8.7 in PCa group while this value was found as 20%±9.3 in the other group (p=0.72).

**Table 1:** The comparison of age, tPSA and fPSA levels, f/tPSA ratio, and prostate volumes of the groups.

	PCa (n=78)	PCa + NIH IV prostatitis (n=23)	p value
Age (years)	66 (43–76)	67 (59–76)	0.45
tPSA (ng/mL)	6.5 (0.6–20)	7.4 (3.5–20)	0.67
f PSA (ng/mL)	0.95 (0.08–3.8)	0.88 (0.31–2.67)	0.78
f/t PSA	0.14 (0.03–0.29)	0.13 (0.04–0.21)	0.32
PV (mL)	35 (15–98)	40 (20–90)	0.031*
PSAD	0.20 (0.02–0.69)	0.16 (0.05–0.59)	0.54

**PSAD** = PSA Density;

* = statistically significant.

## DISCUSSION

Eighty-five percent of patients with PCa are diagnosed at>65 years of age (11). PCa is the most frequent visceral malignant neoplasm in adult males (12). After PSA measurement came into use, the incidence of locoregional disease increased and that of metastatic disease decreased (13). Most cases of PCa were diagnosed according to abnormal DRE findings, high PSA levels, or both in the 1980s and early 1990s. However, most cases diagnosed today are clinically nonpalpable (stage T1c). The optimal treatment is chosen according to the stage of the disease. Therefore, for correct staging, it is important to know the factors that affect the parameters used for staging of PCa.

PSA is a serine protease belonging to the human kallikrein-3 group. It is an organ-specific marker that is produced primarily in the luminal epithelial cells of the prostate (14, 15). PSA production in PCa is not excessively higher than that of the normal prostate tissue (16). A high serum PSA level in patients with PCa is considered to be due to disruption of the cellular architecture of the gland (17). Loss of the barrier comprising the basal layer and basal membrane in the normal prostate gland results in leakage of PSA into the circulation. This may also be seen in other diseases of the prostate gland, such as prostatitis. Additionally, procedures such as prostate massage and prostate biopsy cause an increase in PSA level secondary to prostate manipulation (17, 18). Patients with BPH treated by finasteride or other 5-alpha reductase agents show an approximately 50% decrease in their PSA levels. Therefore, we excluded patients with a history of urological operations and those using agents that can affect the PSA level.

Most patients diagnosed with PCa have a localized stage (cT1c) of clinically insignificant cancer; thus, some patients may be overtreated by definitive therapies. Current guidelines recommend active surveillance for young patients who are in the clinically insignificant and low-risk group with a life expectancy of>10 years (4). The inclusion criteria for active surveillance with the low risk of cancer progression are a PSA level≤10ng/mL, biopsy Gleason score≤6, ≤2 positive cores, ≤50% cancerous tissue per biopsy, and a clinical stage of cT1c to T2a. Radical prostatectomy, which is a considerably more invasive procedure, is therefore delayed. Several studies have shown very low progression and cancer-specific death rates with active surveillance performed in well-selected low-risk groups (5-7). Various nomograms have been developed to predict the prognosis of PCa and select the appropriate treatment. The most frequently used and simplest nomogram was developed by D'Amico et al. (3). In this classification system, patients diagnosed with PCa are divided into low-, intermediate-, and high-risk groups according to their preoperative PSA level, clinical stage, and Gleason score. Another frequently used nomogram involves the Epstein criteria. The difference in this classification system is the absence of the PSA level among the criteria and the inclusion of the PSAD. This classification system indicates that the PSAD should be <0.15ng/mL in the low-risk group (19). We also investigated the effect of NIH IV prostatitis on PSAD in the present study and found similar PSADs in both groups (Table-1). This result indicates that NIH IV prostatitis does not affect PSAD.

NIH category IV prostatitis is an asymptomatic inflammatory prostatitis. Such patients may seek treatment for BPH, PCa, high serum PSA levels, or infertility. Inflammatory cells are seen in prostate biopsy specimens, histopathological examination of transurethral resection bites performed due to PCa or BPH, or microscopic examination of expressed prostatic secretion or semen.

Does the presence of NIH IV prostatitis increase the PSA level in patients with localized PCa? In the present study, the median PSA level was higher in the PCa+NIH IV prostatitis group than in the PCa group; however, the difference was not statistically significant (7.4 (3.5–20.) versus 6.5 (0.6–20.0) ng/mL, respectively; p 0.67). This finding was considered to indicate that NIH IV prostatitis accompanying PCa does not cause an additional increase in PSA compared with PCa alone. On the other hand, Aglamis et al. (20) recently evaluated 198 patients with PCa and found that NIH IV prostatitis (Group 2) accompanying PCa was associated with significantly increased PSA levels. However, the PSA range was wider and the study population was more heterogeneous in their study than in this work.

The PSA level increases as the PV increases. Each 1-mL increase in the PV causes a 4% increase in the PSA level (21). Although the median PV was higher in our PCa+NIH IV prostatitis group (40 versus 35mL, respectively; p=0.035), the PSA levels in this group were not different from those in the PCa group.

The absence of significantly higher PSA levels in the PCa+NIH IV prostatitis group in our study indicates that prostatitis accompanying PCa does not cause any additional increase in PSA levels. Therefore, it is not possible to evaluate low-risk patients as intermediate-risk patients.

Limitations of our study include the relatively small number of patients and its retrospective nature. Another limitation is selection bias. Some patients harboring occult low-grade PCa with or without inflammation do not undergo biopsies. On the other hand, the main strength of our study is inclusion of patients with stage cT1c to T2a PCa, a Gleason score≤6, ≤2 positive cores, and ≤50% cancerous tissue per biopsy. Our study is also the first to evaluate the effect of NIH IV prostatitis on PSA levels in patients with low-risk localized PCa.

## CONCLUSIONS

According to our study; NIH IV prostatitis accompanying PCa was not associated with a statistically significant difference in PSA level. Further studies with larger patient cohorts are needed to clarify this issue.

## Abbreviations

BPH: = Benign prostate hypertrophy
DRE: = Digital rectal examination
fPSA: = Free prostate-specific antigen
NCCN: = National Comprehensive Cancer Network
NIH: = National Institute of Health
P**Ca**: = Prostate cancer
PSA: = Prostate-specific antigen
PSAD: = Prostate-specific antigen density
PV: = Prostate Volume
t**PSA**: = total Prostate-specific antigen
TRUS: = Transrectal ultrasonography
